# Estimating effective population size using RADseq: Effects of SNP selection and sample size

**DOI:** 10.1002/ece3.6016

**Published:** 2020-02-11

**Authors:** Florianne Marandel, Grégory Charrier, Jean‐Baptiste Lamy, Sabrina Le Cam, Pascal Lorance, Verena M. Trenkel

**Affiliations:** ^1^ Ifremer Ecologie et Modèles pour l’Halieutique Nantes France; ^2^ Laboratoire des Sciences de l’Environnement Marin (LEMAR, UMR 6539 CNRS/IRD/UBO/Ifremer) Université de Bretagne Occidentale Institut Universitaire Européen de la Mer Plouzané France; ^3^ Ifremer Génétique et Pathologie des Mollusques Marin (SG2M‐LGPMM) La Tremblade France

**Keywords:** effective population size, linkage disequilibrium, NeEstimator, RADseq, skates and rays

## Abstract

Effective population size (*N_e_*) is a key parameter of population genetics. However, *N*_e_ remains challenging to estimate for natural populations as several factors are likely to bias estimates. These factors include sampling design, sequencing method, and data filtering. One issue inherent to the restriction site‐associated DNA sequencing (RADseq) protocol is missing data and SNP selection criteria (e.g., minimum minor allele frequency, number of SNPs). To evaluate the potential impact of SNP selection criteria on *N_e_* estimates (Linkage Disequilibrium method) we used RADseq data for a nonmodel species, the thornback ray. In this data set, the inbreeding coefficient *F*_IS_ was positively correlated with the amount of missing data, implying data were missing nonrandomly. The precision of *N_e_*estimates decreased with the number of SNPs. Mean *N_e_* estimates (averaged across 50 random data sets with2000 SNPs) ranged between 237 and 1784. Increasing the percentage of missing data from 25% to 50% increased *N_e_* estimates between 82% and 120%, while increasing the minor allele frequency (MAF) threshold from 0.01 to 0.1 decreased estimates between 71% and 75%. Considering these effects is important when interpreting RADseq data‐derived estimates of effective population size in empirical studies.

## Graphical abstract

SNP‐based estimates of effective population size were found to be sensitive to SNP filtering threshold values. Increasing the percentage of missing data from 25% to 50% increased Ne estimates between 82% and 120%, while increasing the minor allele frequency (MAF) threshold from 0.01 to 0.1 decreased estimates by around 70%. Sample size had a small effect beyond a minimum number.

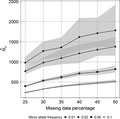

## 1 INTRODUCTION

Effective population size (*N_e_*) is a valuable parameter in population genetics and conservation (Hamilton, 2009; Hare et al., 2011). This parameter is related to the number of individuals which actually participate to produce the next generation and thus informs on population viability (Soulé, 1987). However, estimating *N_e_* can be challenging. Theoretically from a genetic point of view, *N_e_* is defined as the size of an ideal population that would experience the same rate of change in allele frequencies or heterozygosity as the observed population (Beaumont, Boudry, & Hoare, 2010; Hamilton, 2009; Wright, 1931). Ideal populations are constituted of diploid organisms with sexual reproduction, nonoverlapping generations, random mating, no migration, no mutation, but also no natural selection and constant population size (Wright, 1931); census population size is equal to effective population size in an ideal population.

Two main approaches are employed for estimating *N_e_*: demographic methods based on life history traits and genetic methods based on genetic markers. Demographic approaches estimate *N_e_* as a function of parameters such as mean and variance in offspring number, survival‐at‐age, and birth rate. However, demographic methods often rely on strong assumptions such as discrete generations (Caballero, 1994; Nomura, 2002) or if overlapping generations are admitted, stable age structure (Robin S. Waples, Do, & Chopelet, 2011). Only one demographic method allows demographic stochasticity and heterogeneity at the expense of challenging data demands such as individual‐level information (Engen, Lande, Saether, & Gienapp, 2010). This might explain why the method has not been much used so far (but see Trask, Bignal, McCracken, Piertney, & Reid, 2017).

Genetic methods have gained in popularity and power due to recent advances in genotyping and sequencing technologies as well as in computer processing speed. They rely on the extraction of genetic signals (allele frequencies) which are theoretically known to be affected by population demography, mainly effective population size. Among the genetic methods available, single‐sample approaches are appealing since they require sampling only at one point in time. The most popular *N*_e_ estimator is based on a measure of linkage disequilibrium (LD), that is, the nonrandom association of alleles at different loci. The LD method has been widely used during the last decade for a variety of organisms, including mammals (Cervantes, Pastor, Gutiérrez, Goyache, & Molina, 2011; Juarez et al., 2016), insects (Francuski & Milankov, 2015), reptiles (Bishop, Leslie, Bourquin, & O’Ryan, 2009), and fishes (Pilger, Gido, Propst, Whitney, & Turner, 2015; Wilson, McDermid, Wozney, Kjartanson, & Haxton, 2014).

Empirical estimates of *N_e_* are often biased because all methods rely on strong assumptions which are likely violated in natural populations (R. S. Waples, Antao, & Luikart, 2014). Numerous recent genetic studies have documented how more realistic simulations or real data, which do not fulfill methods’ assumptions, lead to biased *N_e_* estimates (Gilbert & Whitlock, 2015; Hare et al., 2011; Luikart, Ryman, Tallmon, Schwartz, & Allendorf, 2010; Marandel et al., 2019; Robinson & Moyer, 2013; Russell & Fewster, 2009; R. S. Waples et al., 2014; Robin S. Waples & Do, 2010). Among the various sources of bias, the assumption of nonoverlapping generations is often violated. In this case, the amount and the direction of bias as well as the precision of estimates are highly dependent on life history traits, thus species‐specific, but also on the sampling fraction (Marandel et al., 2019; R. S. Waples et al., 2014). Other factors such as unequal sex ratio, high level of inbreeding and high variance in family sizes have also been found to bias *N*_e_ estimates(Montarry et al., 2019).

For nonmodel species, the absence of a reference genome challenges the development of genetic markers and the assessment of genomic ascertainment bias, and more generally the amount of expected species‐specific bias for *N_e_* estimates. A widely used method to develop de novo genetic markers and genotype individuals in one single step is the restriction associated DNA sequencing (RADseq), which provides thousands of sequenced SNP (single‐nucleotide polymorphism) markers across many individuals at reasonable costs (Davey & Blaxter, 2010). A drawback of the method is the numerous sources of genotyping errors (Mastretta‐Yanes et al., 2015) and missing data (information missing for certain individuals for certain markers). One case of genotyping errors is dropped alleles, that is, one allele is not typed making a heterozygous individual appearing homozygous (Bilton et al., 2018). Missing data and random allelic dropouts can bias LD estimates (Akey, Zhang, Xiong, Doris, & Jin, 2001; Bilton et al., 2018) and subsequently bias LD based *N*_e_ estimates and increase their variance (Nunziata & Weisrock, 2018; Russell & Fewster, 2009). The degree of bias in LD estimates depends on allele frequency (Akey et al., 2001). Further, rare alleles are known to cause positive bias in *N*_e_ estimates (e.g., Nunziata & Weisrock, 2018; Russell & Fewster, 2009). For microsatellites, this has led to the recommendation to select those with minor allele frequency (MAF) >0.01 if sample size >100 (Robin S. Waples & Do, 2010). For SNPs, rare alleles can be avoided by keeping only the SNPs with highest polymorphic content (Phillips et al., 2004). However, the effect of the MAF threshold remains poorly known, but see Nunziata and Weisrock (2018).

**Figure 1:**
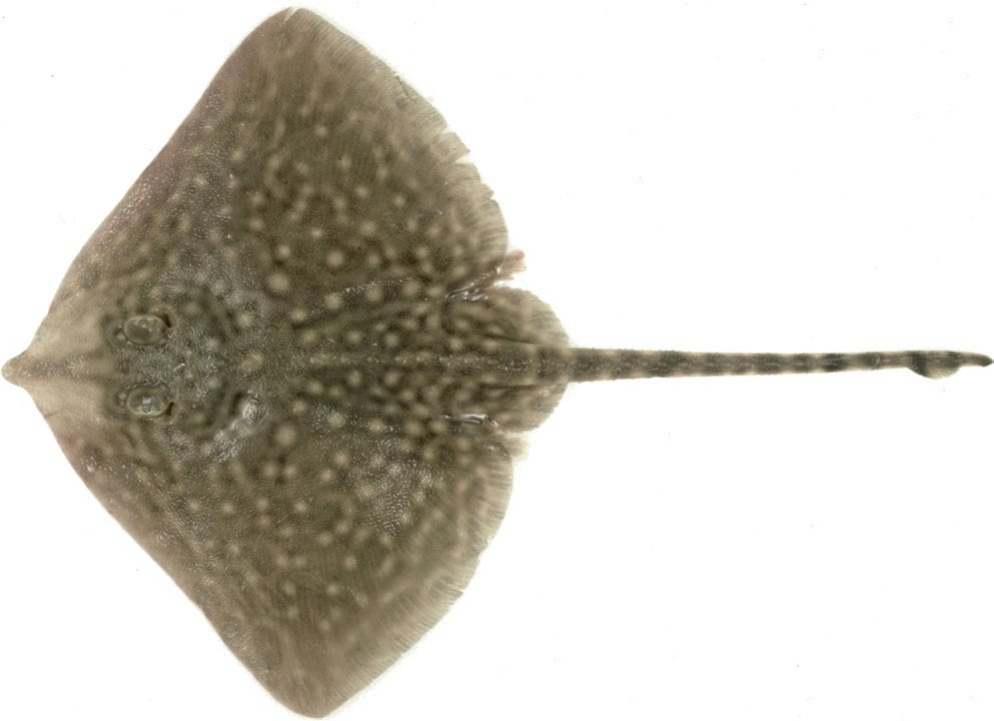
Thornback ray *Raja clavata*

The aim of this study was to determine the effects of the MAF, the proportion of missing data and the number of SNPs on *N_e_* estimates when applying the LD approach to RADseq data. These effects were explored using empirical data collected for the thornback ray (*Raja clavata,* Figure 1) in the Bay of Biscay.

## 2 MATERIAL AND METHODS

### 2.1 Sampling

**Figure 2:**
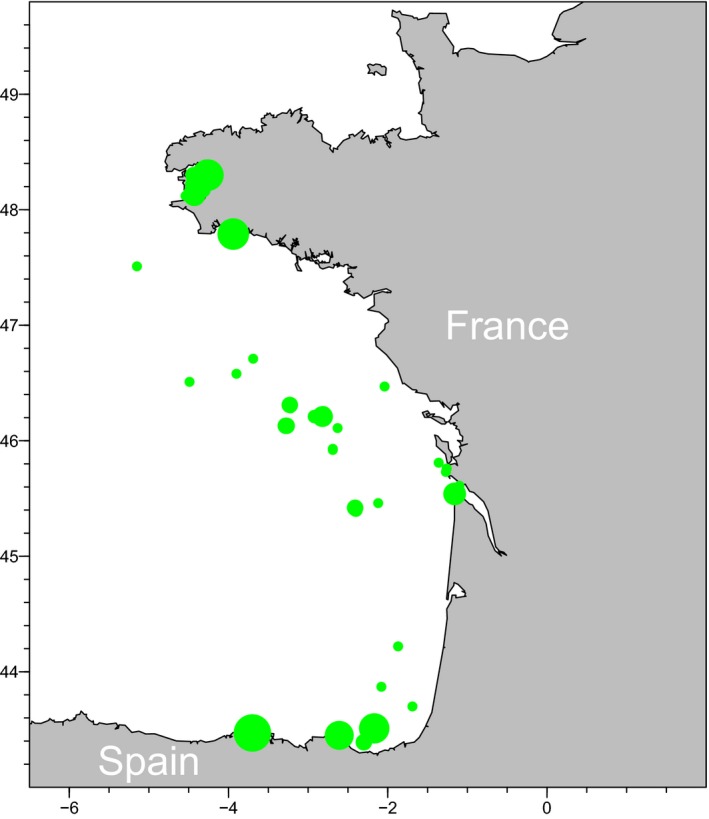
Sampling locations of thornback rays in the Bay of Biscay. Number proportional to bubble surface

Overall 159 thornback rays were sampled in the Bay of Biscay between 2011 and 2016 (half the samples were collected in 2015) (Figure 2). Sampled individuals were collected at sea (EVHOE and RaieJuve surveys carried out by Ifremer) and at landing ports from commercial fisheries. The sex ratio of the sample was close to 1:1 (78 females, 81 males). Total length varied from 12.5 to 96 cm.

### 2.2 RAD‐sequencing protocol and bioinformatics

All individuals were genotyped by sequencing using a RADseq protocol to effectively subsample the genome of multiple individuals at homologous genomic regions. The library construction followed the original protocol by Baird et al. (2008) with slight modifications. Briefly, 1 μg of genomic DNA from each individual was digested with the restriction enzyme *Sbf*I‐HF (New England Biolabs), and then ligated to a P1 adapter labeled with a unique barcode. We used 16 barcodes of 5‐bp and 16 barcodes of 6‐bp length in our P1 adapters to build 32‐plex libraries. The 159 individuals were part of a wider sample including individuals from other regions. From these, one pool of three individuals and seven pools of 32 individuals were made by mixing individual DNA in equimolar proportions and sheared to an average size of 500 and 350 bp, respectively, using a Covaris S220 sonicator (KBiosciences). A size‐selected step was carried out on agarose gel to keep DNA fragments within the size range 500–1000 bp for the 3‐plex and 300–700 pb for the 32‐plexes. Each library was then submitted to end‐repair, A‐tailing, and ligation to P2 adapter before PCR amplification for 18 cycles. Amplification products from six PCR replicates were pooled for each library, gel‐purified after size selection and quantified on a 2,100 Bioanalyzer using the High Sensitivity DNA kit (Agilent). The 3‐plex library was sequenced in paired‐ends 300 reads using Illumina Miseq technology. Each 32‐plex library was sequenced on a separate lane of an Illumina Hiseq 2500 instrument by INTEGRAGEN, using 100‐bp single reads.

We aligned *ca* 54.6M paired‐end Miseq reads to the little skate (*Leucoraja erinacea*) genome assembly (Wang et al., 2012; Wyffels et al., 2014) using BWA‐SW (version 0.7.12‐r1039, default parameters) to build up thornback ray consensus sequences from high quality mapped reads (mapQ score = 60). The result was used as a reference for further analyses. Raw sequences from the 32‐plexes were quality checked, trimmed to 95bp and demultiplexed using the process_radtags module of Stacks v1.32(Catchen, Hohenlohe, Bassham, Amores, & Cresko, 2013). Demultiplexed sequences were aligned to the custom reference genome from the mapped Miseq reads using BWA‐SW version 0.7.12‐r1039 (default parameters) for locus assembly and SNP calling was achieved with the reference mapping pipeline ref_map.pl (Stacks v1.32; (Catchen et al., 2013). Individuals were genotyped on 389 483 putative SNPs spread on 35 134 RAD loci (a sequence starting or ending with a restriction enzyme site). Given the variability due to laboratory work and sequencing protocols, we chose to retain only the loci with a calling rate percentage above 50% (i.e., maximum percentage of missing data (NA) of 50%) and a MAF above 0.01 for the 159 individuals to remove spurious SNPs. This raw data set had 43 088SNPs (Le Cam et al., 2019). Given the large number of amplification cycles (18), a preliminary analysis of the dependence of the heterozygote miscall rate on mean SNP read depth was carried out using the R package whoa (Anderson, 2019) as suggested by a reviewer. The potential heterozygote miscall rate was estimated from comparing the observed number of heterozygous individuals with the number expected given the allele frequency and assuming the SNP was in Hardy–Weinberg equilibrium (Hendricks et al., 2018). Based on this a data set containing only genotypes with read depths between 30 and 300 copies, MAF ≥0.01 and NAs ≤0.5 was created (referred to as full data, 17 843 SNPs). Removing genotypes with low and very high read depths (below 30 and above 300 copies) reduced the number of SNPs but also increased the proportion of missing data. A second data set with a maximum NA of 25% was therefore created (referred to as reduced data, 4,816 SNPs). The lower NA threshold value is more in line with common practices in empirical studies (e.g., 15% missing data in Pazmino, Maes, Simpfendorfer, Salinas‐de‐Leon, and van Herwerden (2017), 25% in Rodriguez‐Ezpeleta et al. (2016)).

The randomness of missing data in the full data set was tested by estimating the Spearman rank correlation between the proportion of missing data and the inbreeding index *F*_IS_ = 1−*H*_obs_/*H*_exp_, where *H*_obs_ is the observed proportion of heterozygous individuals and *H*_exp_ the expected proportion under Hardy–Weinberg equilibrium for a given SNP. We also tested the correlation between the proportion of missing data and the proportion of heterozygous individuals (*H*_obs_).

Seven individuals including five from outside the Bay of Biscay were genotyped twice. This replicate data set was used to explore genotyping error and allelic dropout. Allelic dropout corresponded to one of the replicate genotypes being heterozygous but not the other one or one being homozygous for the major allele and the other for the minor allele. The correlation between the proportion of replicated individuals exhibiting dropout and the inbreeding coefficient for all 159 individuals was tested.

### 2.3 Effective population size

The single point estimation method linkage disequilibrium (LD) is based on linkage disequilibrium due to the nonrandom association of alleles at different gene loci. LD is measured at one point in time by the covariance between loci. We used NeEstimatorV2.1 (Do et al., 2014) for estimating effective population size ${\hat{N}}_{e}$. All samples from different years and cohorts (size classes) were pooled for estimation.

First, the effect of the number of SNPs was evaluated by drawing randomly (without replacement) 500 to 4,000 SNPs from the reduced data set with MAF ≥0.01 and percent missing data NA ≤25%. Fifty replicate data sets were created, and the mean and coefficient of variation (standard deviation/mean) of replicate ${\hat{N}}_{e}$ estimates were calculated.

The effects of four thresholds for the minimum MAF were then evaluated for the full data set: 0.01, 0.02, 0.05, and 0.1 where a value of 0.01 means that the selected SNPs had a MAF in the range 0.01 to 0.5. This rather wide range of threshold values was chosen to explore the shape of the relationship between the MAF filter and ${\hat{N}}_{e}$ estimates. High threshold values (>0.05) might be unsuitable for practical applications (see discussion). The MAF filter was combined with a filter for NA for each SNP which had six levels between 25% and 50% (5% steps). Combining MAF and NA thresholds led to 24 empirical genetic datasets for which ${\hat{N}}_{e}$ was estimated. The number of available SNPs varied between data sets from 1549 to 17 842 (Table 1). For standardization, 2000 SNPs were randomly selected (without replacement) from each data set, except for the smallest data set for which it was 1,000 (NA ≤25%, MAF ≥0.1). This number of SNPs was sufficient to stabilize estimates (see results). An ANOVA was fitted to replicate log‐transformed ${\hat{N}}_{e}$ estimates for comparing the effects of MAF and missing data filters, as well as their interaction. Residuals were checked for normality.

**Table 1:** Number of SNPs available for different data selection thresholds for minor allele frequency (MAF) and missing data

Missing data (%)	MAF lower threshold			
	0.01	0.02	0.05	0.1
25	4,816	3,849	2,374	1549
30	7,072	5,718	3,497	2,238
35	9,388	7,620	4,751	3,030
40	11,913	9,682	5,979	3,754
45	14,782	11,958	7,368	4,566
50	17,842	14,315	8,788	5,401

The effect of the sample size on ${\hat{N}}_{e}$ estimates was evaluated with the reduced data set (MAF ≥0.01; NA ≤25%) by creating random data sets with the number of individuals ranging from 25 to 150. A rarefaction curve analysis was calculated as a function of sample size. A parametric model (Michaelis‐Menten) was fitted using nonlinear least‐squares to estimate ${\hat{N}}_{e}$ free of sample size effects, which corresponds to the model asymptote.

All data handling and analysis of results were carried out in R (R Development Core Team, 2008).

## 3 RESULTS

### 3.1 Exploratory analysis

**Figure 3:**
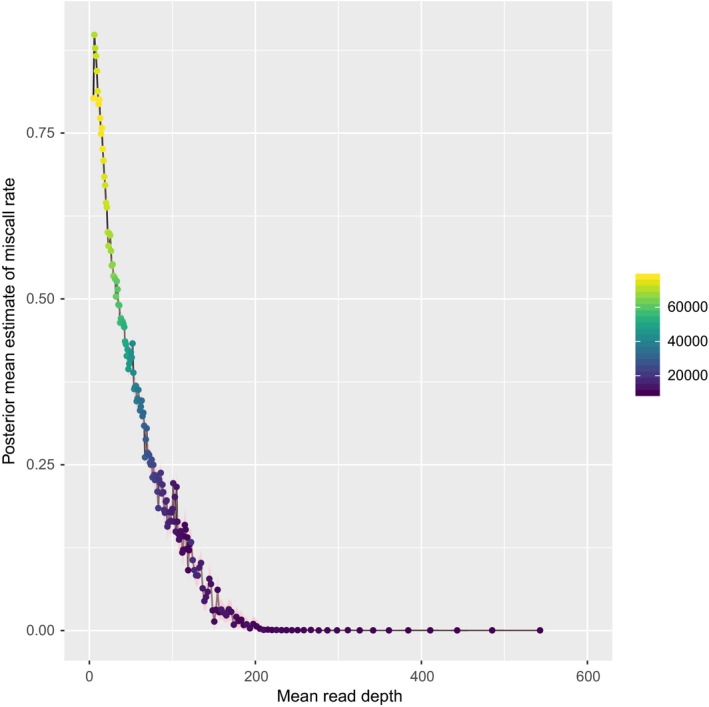
Estimated miscall rate of SNPs as function of mean read depth of each SNP for raw data set for thornback ray in the Bay of Biscay. The color scale indicates the number of data points (number of individuals * number of SNPs)

In the raw data, the estimated mean miscall rate decreased strongly as the minimum read depth increased (Figure 3). It was around 0.75 considering all SNPs in the raw data set. Therefore, further analyses were restricted to genotypes with read depth in the range 30 to 300 copies (full data set).

**Figure 4:**
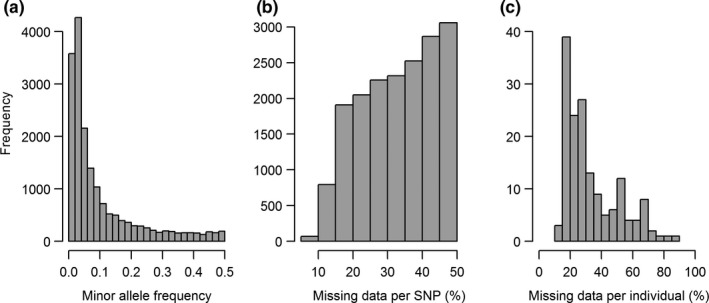
(a) Histogram of minor allele frequencies of SNPs with percentage missing data ≤50%. (b) Histogram of percent missing data for SNPs with minor allele frequency ≥0.01. (c) Percent missing SNPs per individuals for percentage missing data ≤50% and minor allele frequency ≥0.01

The distribution of MAF values in the full data set was nonuniform with most of the SNPs displaying MAF <0.1 (Figure 4a) and NA >25% (Figure 4b). The distribution of missing individuals was nonuniform across individuals with 33 individuals missing more than 50% of SNPs (Figure 4c).

**Figure 5:**
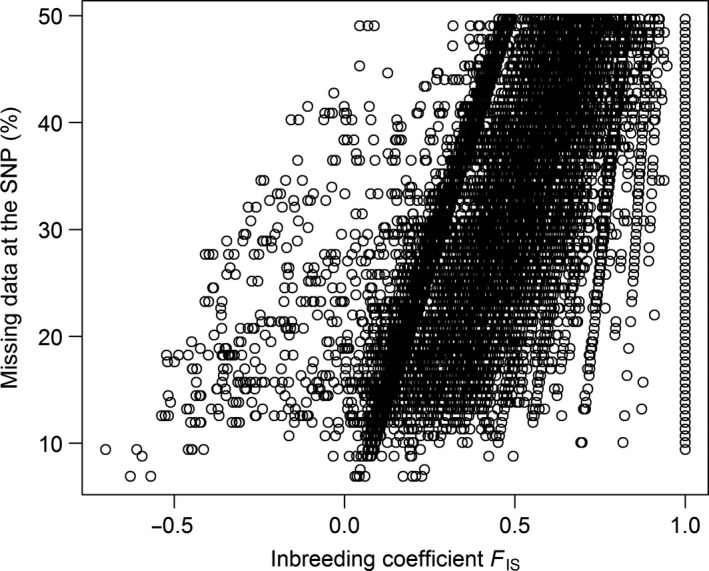
Relationship between the missing data threshold and the inbreeding coefficient of selected SNPs (minor allele frequency ≥0.01; percent missing data ≤50%) for thornback ray in the Bay of Biscay

A significant positive correlation (Spearman's rho = 0.61, *p*‐value < .001) was found between the percentage of missing data and the inbreeding coefficient *F*_IS_ of a given SNP (Figure 5), while the correlation between the proportion of missing data and the proportion of heterozygous individuals was significantly negative (Spearman's rho = −0.28, *p*‐value < .001). Thus, data were not missing at random: Individuals with missing data were more likely to be heterozygous.

For the seven individuals genotyped twice, on average 11% of SNPs (all SNPs genotyped twice) had a different genotype (median 8%, range 4%–19%).When genotypes differed, in 85% (median 89%, range 67%–98%) of cases one replicate was heterozygote and the other homozygote. Further, the proportion of individuals exhibiting allelic dropout for a given SNP was significantly negatively correlated with the inbreeding coefficient for all individuals for the same SNP (Spearman's rho = −0.12, *p*‐value < .001, *n* = 17 842 SNPs).

### 3.2 Ne estimation

**Figure 6:**
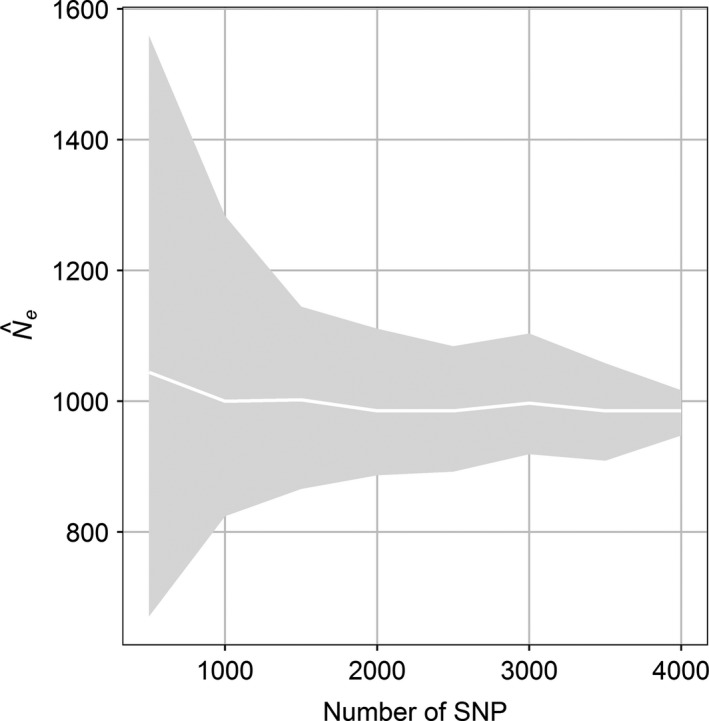
Relationship between Ne estimates and the number of SNPs for thornback ray in the Bay of Biscay (minor allele frequency ≥0.01; percent missing data ≤25%). White line is mean of 50 random data sets and shaded area central 90% percentile band

The mean estimate of ${\hat{N}}_{e}$ across the 50 random data sets stabilized at around 1,500 SNPs and uncertainty decreased with the number of SNPs (Figure 6). The coefficient of variation (CV) decreased from 0.29 for 500 SNPs to 0.07 for 2,000 SNPs and 0.02 for 4,000 SNPs. This indicates that the 2000 SNPs used for exploring the effects of missing data and MAF thresholds were sufficient for obtaining reliable estimates.

**Figure 7:**
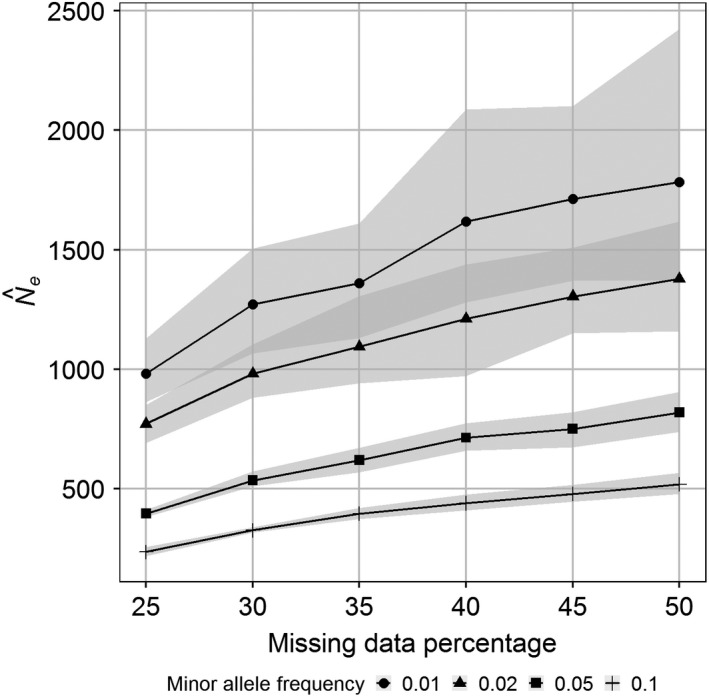
Relationship between Ne estimates and missing data percentage threshold for different threshold levels of the minor allele frequency for thornback ray in the Bay of Biscay. Continuous lines are mean values for 50 random data sets with 2000 SNPs and shaded areas central 90% percentile bands

The effects on ${\hat{N}}_{e}$ estimates of the thresholds for MAF and NA were important (Figure 7). Mean ${\hat{N}}_{e}$ estimates ranged between 237 and 1,784 corresponding to a factor of 7.5. Mean values decreased by 71% to 75% with increasing MAF threshold and increased by 82% to 120% with NA. For example, for the smallest NA (25%), mean ${\hat{N}}_{e}$ (averaged across 50 replicates) decreased by 76% from 982 to 237 as the MAF threshold increased from 0.01 to 0.1. In contrast, for the smallest MAF threshold (0.01), the mean ${\hat{N}}_{e}$ increased by 82% from 982 to 1784 when NA increased from 25% to 50%.

The ANOVA revealed that the effect of the MAF threshold value was eight times larger than that of NA (Table 2). There was a weak but significant interaction between the two factors.

**Table 2:** Analysis of variance for testing the effects of threshold values for percent of missing data (NA) and minimum minor allele frequency (MAF) on log‐transformed effective population size (*N_e_*) estimates

Name	*df*	MS	F	*P*‐value
NA	5	12.21	1,590.25	<.001
MAF	3	101.45	13,215.66	<.001
NA:MAF	15	0.08	10.73	<.001
Residuals	1,176	0.01		

**Figure 8:**
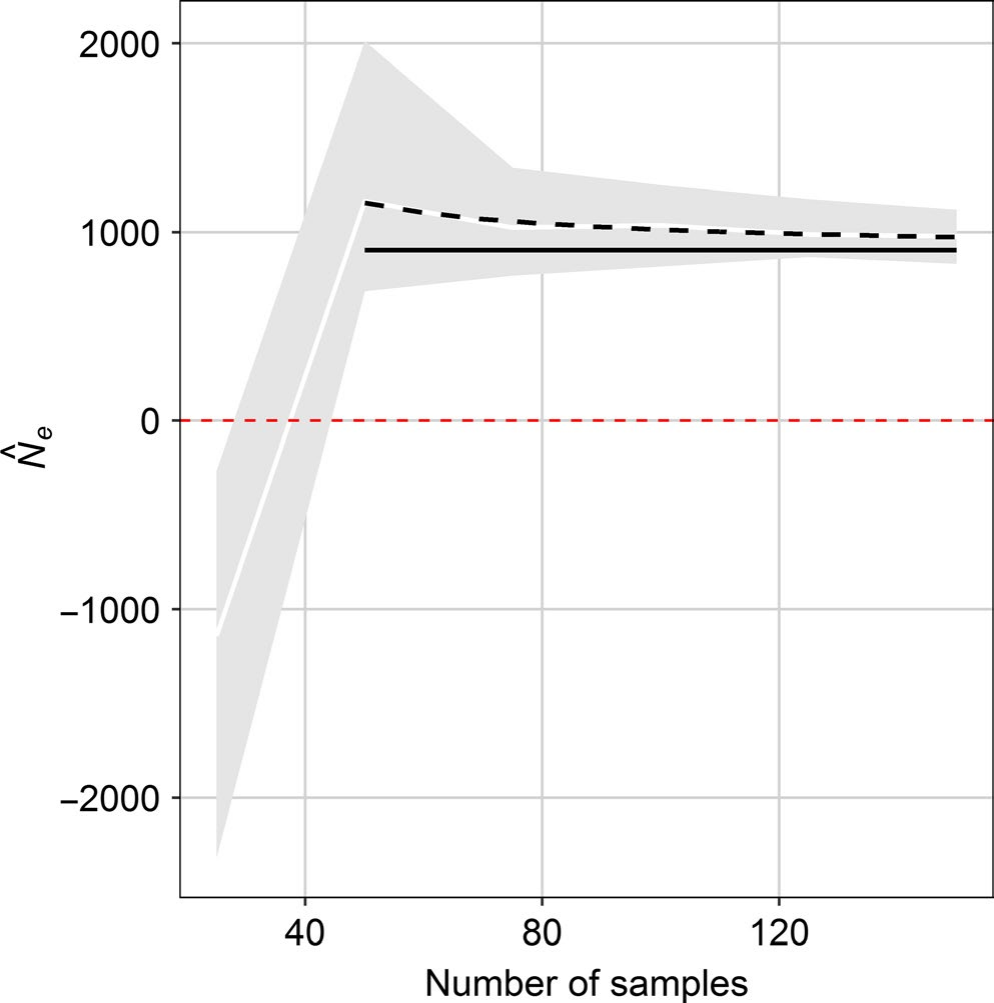
Relationship between Ne estimates and sample size for thornback ray in the Bay of Biscay (minor allele frequency ≥0.01; percent missing data ≤25%). Continuous white line is mean value for 50 random data sets with 2000 SNPs and shaded areas central 90% percentile bands. Black dotted line is fitted model whose asymptote is plotted as continuous horizontal black line

Estimates of *N*_e_ were negative for a sample size of 25 individuals and decreased somewhat from an average of 1,165 for 50 individuals to 977 for 150 individuals (Figure 8). The asymptote of the fitted model ignoring negative estimate was 903 (SE 21.6) which can be interpreted as the estimate that would have been obtained with a sufficient sample size, implying that the 159 individuals were insufficient.

## 4 DISCUSSION

Empirical genetic data from thornback rays sampled in the Bay of Biscay were used to explore the effects of data selection on *N_e_* estimates. Genetic markers were obtained from a RADseq protocol in which individuals with missing data for a given SNP are common (Nunziata & Weisrock, 2018) and genotyping errors frequent (Mastretta‐Yanes et al., 2015). In the thornback ray data, for 11% of SNPs the genotype differed between the two replicates on average across the seven individuals genotyped twice retaining only genotypes with read depth 30–300 copies. Unfortunately, genotyping errors for RADseq data are seldom reported in the literature. Higher disagreement rates have been found for oyster (J.B. Lamy pers. comm.) while lower error rates (2%–12%) have been reported for the plant *Berberis alpina* (Mastretta‐Yanes et al., 2015). Further, SNPs were missing nonrandomly with the amount of missing data increasing with the inbreeding coefficient while, the proportion of replicate individuals with allelic dropout was lower for SNPs with higher inbreeding coefficient. For microsatellites Soulsbury, Iossa, Edwards, Baker, and Harris (2007) also found a relationship between allelic dropout and departure from Hardy–Weinberg equilibrium, that is, the inbreeding coefficient.

Contrary to other ecological studies of nonmodel species a reference genome was used here (assembly from a related species) that allowed us to identify SNPs with greater power and avoid common problems encountered with the *de novo* RADseq analysis such as merging paralogous loci as alleles (e.g., Diaz‐Arce & Rodriguez‐Ezpeleta, 2019).

The uncertainty of *N_e_* estimates obtained with the linkage disequilibrium method decreased strongly with the number of SNPs. Mean values stabilized at around 1,500 SNPs for the full data set. In comparison, Pazmino et al. (2017) used 8,103 neutral SNPs (MAF ≥0.02; missing data ≤15%; replication error ≤5%) for a shark species while Montes et al. (2016) used 349 neutral SNPs for estimating effective population size for an anchovy population and Diaz‐Arce and Rodriguez‐Ezpeleta (2019) 96 SNPs (missing data ≤9%) for salmon.

Subsampling the data with different thresholds for missing data and minimum minor allele frequency permitted us to evaluate the effects of these two factors on *N*_e_ estimates. Depending on the combination of threshold values, *N_e_* estimates varied by up to a factor of 7.5. In comparison, the well‐known effect of underestimation of *N_e_* due to ignoring overlapping generations is only around 30% in the thornback ray, independent of the census population size (Marandel et al., 2019). Further, the MAF threshold value (tested range 0.01 to 0.1) had a larger effect compared with the NA (tested range 25 to 50%). This is not surprising given NeEstimator accounts for missing data (NeEstimator V2.1 online documentation at http://www.molecularfisherieslaboratory.com.au/neestimator-software). The effect of the polymorphism on *N_e_* estimates using LD was previously addressed by Russell and Fewster (2009) for ideal populations, and we agree with these authors that researchers should be aware of the effects of the MAF threshold applied for SNP selection.

In contrast to SNP selection criteria, the sample size was found to impact estimates only slightly, given at least 50 individuals were used. Negative estimates are expected when sample size is insufficient (Marandel et al., 2019). Further, the 159 individuals were probably not enough to obtain stabilized estimates. This might not be surprising given that simplified genetic simulations for a thornback ray like species indicated that around 1% of the population needed to be sampled to obtain reliable estimates (Marandel et al., 2019) and the Bay of Biscay population is potentially large (Marandel, Lorance, & Trenkel, 2016). The rarefaction analysis with the reduced data set indicated a stabilized effective population size of 903 (asymptote of fitted model). For the thornback ray population in the Irish Sea and Bristol Channel Chevolot, Ellis, Rijnsdorp, Stam, and Olsen (2008) estimated *N*_e_ as being 283 using five microsatellites and a temporal estimation method with samples from two time periods. Given the difference in approach, it is unknown whether their sample of 363 individuals and number of microsatellites was sufficient and hence whether the two effective population size estimates can be compared. If they are comparable, the Bay of Biscay populations would be the larger one.

Other genetic simulations for thornback ray populations in European waters using contrasted assumptions for migration rates suggested a stable large scale population structure with little exchange (Marandel et al., 2018). This could mean that migration might not be expected to impact much allele frequencies in European thornback ray population, hence effective population size estimates. Selection can also cause nonrandom association of alleles within and across loci which will again be interpreted as genetic drift (underestimation of *N_e_*) by the linkage disequilibrium estimator (Waples & Do, 2010). Contrary to genetic drift that affects all loci in the genome, selection only affects certain loci (depending on the genetic architecture) but its effect should be diluted when using a large number of SNPs (»100) as done here). Depending on the genetic determinism of the selected traits (monogenic to polygenic) and the intensity of the selective process, the effect on *N*_e_ estimates is hard to predict.

Further, physically unlinked SNPs were assumed, which is clearly unrealistic (Waples, Larson, & Waples, 2016). The number of truly independent SNPs is equal to the number of chromosomes, which is 98 for thornback ray (Nygren, Nilsson, & Jahnke, 1971) times the average number of crossing‐over per chromosome in thornback ray. The finite number of chromosomes will create linkage disequilibrium (more precisely gametic linkage disequilibrium) purely due to physical linkage between SNPs, rather than true *N_e_* changes (Waples et al., 2016).

Based on our results as a guide for practitioners we recommend to use the lowest feasible percentage of missing data, though the precise threshold value will depend on the overall sample size and the expected effective population size. The main principle is to maintain a sufficiently large sample size (in terms of genotyped individuals) for all SNPs included in the analysis. It is more difficult to make recommendations regarding the threshold value for the minor allele frequency. It is important to keep in mind, that in a perfect Fisher‐Wright population, thresholds on MAF values are nonsense since any filtration will remove important genetic information to infer *N_e_*. However, empirical datasets will always contain loci with alleles of spurious low or very low frequencies. There are a growing number of methods to discard spurious SNPs with a low MAF within the bioinformatics pipeline by taking conservative filters on minimum read depth of the loci. Here, a read depth of at least 30 was required to reduce replication error. At the least, the lowest possible MAF filter should be chosen (compromise between loosing relevant genetic information and noise) and the results for different threshold values should be compared.

In conclusion, for nonmodel species special attention should be paid to the interpretation of *N_e_* estimates as large bias in estimates might occur when using the LD method. For thornback ray, we found that nonrandomly missing data, allele frequency filters and sample size had much larger effects than the expected bias due to ignoring overlapping generations (Marandel et al., 2019). We expect these findings to hold for other nonmodel species though we recommend further studies to confirm this.

## AUTHOR CONTRIBUTIONS

GC, PL, and VT designed research, SL carried out laboratory work and bioinformatics, FM and VT analyzed the data and wrote the first draft, all authors critically revised the manuscript.

## Data Availability

Data were be archived at Seanoe https://doi.org/10.17882/70648.
